# In Vitro Evaluation of Combination Therapy with Doxorubicin and Quercetin for Uveal Melanoma

**DOI:** 10.3390/cimb48060636

**Published:** 2026-06-18

**Authors:** Petra Fodor, Barbara Zsebik, Ferenc Fenyvesi, Zsuzsanna Szabó, Anna Vass, Gábor Halmos

**Affiliations:** 1 Department of Biopharmacy, Faculty of Pharmacy, University of Debrecen, 4002 Debrecen, Hungary; zsebik.barbara@pharm.unideb.hu (B.Z.); szabo.zsuzsanna@pharm.unideb.hu (Z.S.); vass.anna@pharm.unideb.hu (A.V.); 2 Doctoral School of Pharmaceutical Sciences, University of Debrecen, 4032 Debrecen, Hungary; 3 Department of Molecular and Nanopharmaceutics, Faculty of Pharmacy, University of Debrecen, 4002 Debrecen, Hungary; fenyvesi.ferenc@pharm.unideb.hu

**Keywords:** uveal melanoma, doxorubicin, quercetin, combination therapy, metastasis, invasion

## Abstract

Background: Uveal melanoma is the most common intraocular malignancy in adults with a poor prognosis. Although local therapies are effective, treatment options for advanced disease remain limited. Combination strategies using chemotherapeutic agents and natural compounds, such as quercetin, are in focus for their potential to enhance antitumor efficiency and overcome resistance. Methods: The effects of doxorubicin, quercetin, and their combination were investigated in uveal melanoma cell lines. Cell viability was determined by an MTT assay, and apoptosis and cell cycle distribution by flow cytometry. Invasion assays were performed to evaluate metastatic potential, while modifications in signaling pathways were analyzed by Western blotting and qPCR. Results: Both doxorubicin and quercetin significantly reduced cell viability and induced apoptotic and necrotic cell death. The combination treatment demonstrated additional inhibitory effects in both cell lines, shown by increased SubG1 populations, reduced invasive capacity, and modulation of signaling pathways. Cell cycle analysis indicated treatment-induced growth inhibition. Notably, pathway modifications varied between cell lines, suggesting heterogeneous responses. Conclusions: Quercetin may potentiate certain antitumor effects of doxorubicin in uveal melanoma, particularly by reducing post-treatment invasiveness and modulating certain PI3K/AKT pathway-related proteins. These results support the possibility of quercetin-based combination therapies, although further molecular and in vivo studies are required.

## 1. Introduction

Uveal melanoma (UM) is a highly aggressive malignancy originating from melanocytes located within the uveal tract of the eye, which includes the iris, ciliary body, and choroid. UM represents for approximately 3–5% of all melanoma cases worldwide, with an annual incidence rate of 5–6 cases per million people, and with a median age at diagnosis of approximately 62 years [1,2].

Uveal melanoma is characterized by common mutations in GNAQ and GNA11, which contribute to cell growth, metastasis and tumorigenesis. In addition, loss of BAP1 expression is associated with metastatic progression, aggressive behavior and a poor prognosis [2,3]. In the present study, two distinctive cell lines were investigated; MEL-202 was used as a primary UM model, while MM28 represented a metastatic UM model displaying BAP1 deficiency, a hallmark of aggressive disease.

After local therapy, UM has low rates of primary tumor recurrence; however, up to 50% of patients develop metastases at distant sites, mostly in the liver [2]. Current treatment methods for advanced UM remain ineffective at preventing metastatic spread, while conventional chemotherapy shows limited clinical benefits due to the intrinsic resistance of UM cells, low response rates, and the complex molecular background of the tumor. In particular, activation of survival-related signaling pathways, including PI3K/AKT and MAPK pathways, may contribute to therapy resistance and disease progression. Therefore, the development of novel therapeutic approaches and combination strategies remains an important objective in UM research [1,2,3].

Doxorubicin (DOX) is an anthracycline chemotherapeutic agent widely used in the treatment of various types of malignancies, including leukemia, lymphoma and breast cancer. Its antitumor activity is mediated by DNA intercalation and topoisomerase II inhibition, resulting in disrupted DNA replication and transcription in cancer cells. Despite its clinical use, doxorubicin is associated with several limitations, including cardiotoxicity, the development of drug resistance, limited effectiveness in certain tumor types, and the risk of metastatic malignancies. To overcome these complications, there is an urgent need to actively study novel strategies enhancing the therapeutic efficiency of doxorubicin while reducing its adverse effects [4].

Quercetin (QUE) is a naturally occurring flavonoid, found in a variety of plants and fruits, including apples, cherries, broccoli and onions, as well as in dietary sources such as green tea and red wine. It has many biological activities, including anticancer, anti-inflammatory and antioxidant effects. In oncology, quercetin demonstrates considerable potential due to its chemopreventive and therapeutic properties, as seen in both cellular and animal models [5,6,7].

The phosphatidylinositol 3-kinase/protein kinase B (PI3K/AKT) signaling pathway has a central role in regulating cell growth and survival in UM, and is aberrantly activated in over 50% of patients [3]. The PI3K/AKT pathway is closely connected with key regulators of cell survival and apoptosis, including nuclear factor kappa B (NF-κB) [8] and p53 [9]. Additionally, it contributes to the downregulation of cell adhesion molecules, thereby promoting increased motility and assisting the migration of uveal melanoma cells to the liver [10]. Quercetin has been reported to interfere with multiple oncogenic signaling pathways, including PI3K/AKT, consequently promoting apoptosis and inhibiting proliferation in different types of cancer models [5,7]. In addition, it has been shown to modulate oxidative stress and improve the efficacy of common chemotherapeutic agents [11,12]. These advantages make quercetin a candidate for combination therapy approaches aimed at improving anticancer effects.

As other studies demonstrated, combination-based therapeutic approaches may provide greater antitumor efficacy than monotherapies by simultaneously targeting multiple cellular pathways involved in tumor survival, proliferation and metastasis [13,14].

Because UM is resistant to systemic therapies [15,16], this study investigated the growth inhibitory effects of DOX and QUE individually and in combination. We detected changes in PI3K/AKT-related protein expressions, apoptosis, and invasion-related mechanisms.

## 2. Materials and Methods

### 2.1. Cell Lines

The MEL-202 human primary uveal melanoma cell line was purchased from Merck KGaA (Darmstadt, Germany, catalogue number: 13012457-1VL). The MM28 human metastatic uveal melanoma cell line was purchased from ATCC (Manassas, VA, USA, catalogue number: CRL-3295). MEL-202 cells were cultured in complete growth medium RPMI-1640 (LM-1640/500), containing L-glutamine and supplemented with 10% fetal bovine serum (FBS), 100 U/mL penicillin, and 100 μg/mL streptomycin. MM28 cells were cultured in complete growth medium RPMI-1640 (30-2001) containing 2 mM L-glutamine, 10 mM HEPES, 1 mM sodium pyruvate, 4500 mg/L glucose, and 1500 mg/L sodium bicarbonate, supplemented with 20% fetal bovine serum (FBS), 100 U/mL penicillin, and 100 μg/mL streptomycin. UM cell lines were cultured in a humidified chamber (95% air humidity, 5% CO_2_) at 37 °C. All experiments were performed using low-passage numbers (3–5) of MEL-202 and MM28 cells. Routine fluorescence microscopic evaluation following DAPI staining did not indicate signs of mycoplasma contamination during the experiments. All cell lines used in this study were commercially obtained as established human uveal melanoma cell lines.

### 2.2. Chemotherapeutics

Quercetin was purchased from Sigma (St. Louis, MO, USA, catalogue number: Q4951, batch number: SLCP7706, 98% purity), dissolved in DMSO as a vehicle and stored as a stock solution in aliquots at −20 °C. Doxorubicin was purchased from TEVA (Debrecen, Hungary, 2 mg/mL injection solution) and stored at 4 °C. The cells with DMSO exposure served as controls. In the MTT assay, the volume of DMSO was always equivalent to the highest quercetin concentration used in the corresponding experiment.

### 2.3. Cell Viability Assay

The viability of MEL-202 and MM28 UM cells was determined using the colorimetric MTT assay. We seeded 6000 cells/well in a 96-well plate in complete growth media and incubated at 37 °C for 24 h. The next day, the entire media was replaced with culture medium containing DOX (0.01–10 µM) and QUE (5–100 µM) alone and in combination at different concentrations. All types of treatments were performed in triplicate (*n* = 3). The cells were treated for 72 h and incubated at 37 °C in the humidified chamber with 5% CO_2_/95% air humidity. Cell viability was assessed by adding MTT reagent to the medium; the 96-well plates were incubated for 2 h at 37 °C. Afterwards, the media was aspirated from the wells and replaced by 100 µL DMSO, then incubated for 15 min at 37 °C. Absorbance was measured at 560 nm, with 660 nm as the reference in a FLUOstar Optima Counter (BMG Labtech GmbH, Ortenberg, Germany). Results were normalized to the untreated control.

Dose–response curves were constructed, and IC_50_ values were estimated by nonlinear regression using a variable slope model in GraphPad Prism version 8.0. The resulting IC_50_ values were used to define the concentration ranges applied in combination analyses. IC_50_ values, corresponding to drug concentrations reducing cell viability to 50% relative to the control, were derived from three independent biological replicates (*N* = 3).

Based on the calculated IC_50_ values, MEL-202 cells were treated with 1 µM DOX and 50 µM QUE, while MM28 cells were exposed to 1 µM DOX and 70 µM QUE or their respective combination for 72 h. The concentrations used for combination treatments were selected based on preliminary dose–response experiments and IC50 values obtained from MTT assays. Concentrations slightly above the calculated IC50 values were applied to ensure measurable biological effects while maintaining sufficient viable cell populations for analyses, including apoptosis, cell cycle, and invasion. A fixed concentration of 1 µM doxorubicin was used in both cell lines to allow a direct comparison between treatment responses, while quercetin concentrations were adjusted according to the relative sensitivity of the individual cell lines.

### 2.4. RNA Isolation and cDNA Synthesis

Total RNA was isolated from both cell lines using the NucleoSpin RNA/Protein kit (Macherey-Nagel, Düren, Germany), following the manufacturer’s instructions. For complementary DNA (cDNA) synthesis, 1000 ng of RNA was reverse transcribed to cDNA using a Tetro cDNA Synthesis Kit (Bioline, London, UK). The experiment was performed in three independent biological replicates (*N* = 3).

### 2.5. Quantitative Real-Time PCR (qRT-PCR)

qRT-PCR analysis was performed to evaluate the mRNA expression levels of NF-κB1 and p53 in MEL-202 and MM28 cells following treatment with DOX and QUE alone, or their combination. A total of 50 ng of reverse-transcribed cDNA was used for qRT-PCR, performed with specific primers (Supplementary Table S1), using Cyclophilin A (CYC A) as a housekeeping gene. The amplification reactions were performed in 96-well plates using iQ™ SYBR^®^ Green Supermix (Bio-Rad Laboratories, Hercules, CA, USA) according to the manufacturer’s protocol using a CFX-96 Real-Time System (BioRad Laboratories Inc., Hercules, CA, USA) in a 10 μL final reaction volume in technical replicates (*n* = 3). The thermal cycling conditions included an initial annealing step (95 °C for 3 min), followed by 40 amplification cycles (95 °C for 15 s, optimal melting temperature for each primer for 1 min) and melting curve analysis performed from 55 °C to 95 °C in 0.5 °C/15 s. Gene expression was quantified by the 2^−ΔΔCt^ method. To normalize the expression levels of target genes, results were normalized to CYC A. All samples were run in triplicate in three independent biological replicates (*N* = 3).

### 2.6. Protein Isolation

MEL-202 cells were incubated with 1 µM DOX and 50 µM QUE, whereas MM28 cells were exposed to 1 µM DOX and 70 µM QUE, either alone or in combination, for 72 h. After the treatments, the cells were washed with PBS and lysed in M-PER Mammalian Protein Extraction Reagent (Thermo Fischer Scientific, Waltham, MA, USA), supplemented with Protease Inhibitor Cocktail and Phosphatase Inhibitor Cocktail 2 (Sigma-Aldrich, St. Louis, MO, USA) in a ratio of 1:100. The protein concentration was determined by the Bradford assay. The experiment was performed in three independent biological replicates (*N* = 3).

### 2.7. Western Blot

Samples were diluted with 4× Laemmli buffer in dH_2_O and boiled at 100 °C for 5 min. Equal amounts of protein (30 µg) were separated on 10% SDS PAGE and transferred to the PVDF membrane. Membranes were blocked by 5% non-fat dry milk in Tris-buffered saline containing 0.1% Tween-20 for 1 h. Specific antibodies were used to detect the target proteins listed in Supplementary Table S2. Primary antibodies were applied in 1:2000, 1:1000 or 1:500 dilutions. Following incubation with primary antibodies overnight at 4 °C, membranes were incubated for 1 h with horseradish peroxidase (HRP)-tagged anti-rabbit IgG secondary antibody (Thermo Fisher Scientific, Waltham, MA, USA), and bands were detected using Clarity Western ECL Substrate (Bio-Rad Laboratories, Hercules, CA, USA). Densitometric analysis was performed with the ChemiDoc Imaging System using Image Lab Software 5.2 (Bio-Rad Laboratories, Hercules, CA, USA). Target protein expression levels were normalized to the housekeeping protein, Cyclophilin A (Cell Signaling Technology, Danvers, MA, USA). Densitometric values were calculated relative to the untreated control group (control expression = 1). For selected targets, PVDF membranes were stripped, reprobed, or cut for the detection of multiple proteins; therefore, the same loading control was used for all proteins analyzed on the same membrane. All samples were run under identical loading conditions (100 V, 90 min). The experiment was performed in three independent biological replicates (*N* = 3).

### 2.8. Invasion Assay

A cell invasion assay was performed using a QCM^TM^ 96-Well Cell Invasion Assay kit (Merck KGaA, Darmstadt, Germany) according to the manufacturer’s protocol. UM cells were treated with 1 µM of DOX and different concentrations of QUE (50 and 70 μM, respectively) and their corresponding combination for 72 h, and then harvested and seeded in the upper compartment of the invasion chamber. The number of cells that moved into the lower chamber represented the invading cells. Serum-free medium was added in the lower chamber used as the negative control. Invasion was quantified in triplicate following the manufacturer’s instructions. Samples without cells, but containing Cell Detachment Buffer, Lysis Buffer and CyQuant Dye were used as blanks for interpretation of data. The experiment was performed in three independent biological replicates (*N* = 3).

### 2.9. Detection of Early and Late Apoptosis by Flow Cytometry

Apoptotic cell measurement was determined by an Annexin V-FITC Apoptosis Detection Kit II (Merck KGaA, Darmstadt, Germany). MEL-202 and MM28 cells were seeded at 2 × 10^6^ cells into flasks. After overnight incubation, cells were treated differently with DOX (1 µM) and QUE (50 and 70 µM) alone or with their respective combinations. After 72 h, they were harvested, washed with PBS two times and resuspended with 1× binding buffer at a concentration of 5 × 10^5^ cells/mL. Next, 5 μL of AnnexinV-FITC was added and incubated for 10 min, then washed with 1× binding buffer. Next, the pellet was resuspended in 190 μL 1× binding buffer, and 10 μL propidium iodide (PI) was added to the solution. The apoptotic and necrotic cells were detected using Guava easyCyte HT flow cytometry (Merck KGaA, Darmstadt, Germany). Cells in early apoptosis were labeled with Annexin V-FITC only; however, cells in late apoptosis were labeled with Annexin V-FITC and PI too. The experiment was performed in three independent biological replicates (*N* = 3).

### 2.10. Cell Cycle Analysis

MEL-202 and MM28 cells were cultured at 2 × 10^6^ cell density. After overnight incubation, cells were treated differently with DOX (1 µM) and QUE (50 and 70 µM) monotherapy or with their respective combinations. After 72 h, cells were harvested and fixed by 70% EtOH for 15 min on ice, then washed with PBS. Flow cytometry samples were prepared at approximately 1 × 10^6^ density of cells in suspension. Samples were resuspended in 0.5 mL of FxCycle^TM^ PI/RNase Staining Solution, and incubated for 30 min at room temperature, protected from light. Cells were analyzed using Guava easyCyte HT flow cytometry (Merck KGaA, Darmstadt, Germany). The experiment was performed in three independent biological replicates (*N* = 3).

### 2.11. Statistical Analysis

Statistical analyses were performed by one-way ANOVA followed by Tukey’s multiple comparison test in GraphPad Prism 8 software (GraphPad Software Inc., San Diego, CA, USA). A *p* value * < 0.05 was considered to be statistically significant vs. control (*p* value ** < 0.01, *p* value *** < 0.001, *p* value **** < 0.0001). A *p* value × < 0.05 was considered to be statistically significant compared to DOX monotherapy (×× *p* < 0.01, ××× *p* < 0.001, ×××× *p* < 0.0001). A *p* value † *p* < 0.05 was considered to be statistically significant relative to QUE monotherapy (†† *p* < 0.01, ††† *p* < 0.001, †††† *p* < 0.0001).

## 3. Results

### 3.1. Cell Viability and IC_50_ Values

MEL-202 and MM28 human uveal melanoma cell lines were used to investigate the effect of doxorubicin and quercetin on cell viability. Cells were treated with different concentrations of doxorubicin (0.01, 0.1, 1, 5, 10 µM) and quercetin (5, 12.5, 25, 50, 70, 85, 100 µM), and cell viability was analyzed by MTT assay.

MTT assay results demonstrated that treatment with DOX and QUE, either alone or in combination for 72 h, led to a significant reduction in the viability of MEL-202 and MM28 uveal melanoma cells in a dose-dependent manner. In the case of both cell lines, at the end of 72 h incubation, the cell viability of the control group was observed as 100%.

**Figure 1:**
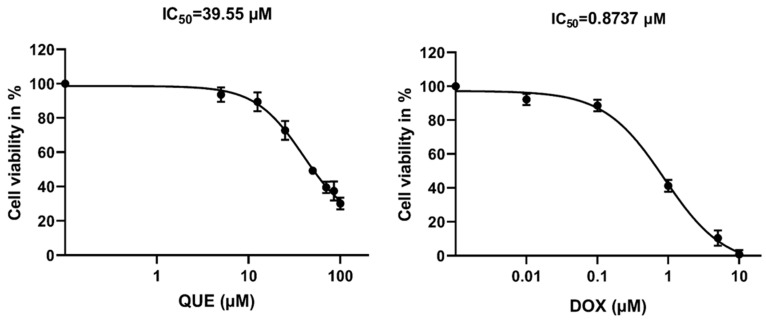
Dose–response curves for QUE and DOX monotherapies in MEL-202 cells following 72 h exposure. The curves were generated based on the MTT assay results, and the IC_50_ values were calculated using nonlinear regression analysis. Percent change represents the reduction in cell viability relative to the control group. Black spots represent individual data points, and the lines indicate the fitted dose–response model.

The calculated half-maximal inhibitory concentration (IC_50_) value of QUE for MEL-202 is approximately 39.55 μM, while for DOX, it is 0.8737 μM (Figure 1).

**Figure 2:**
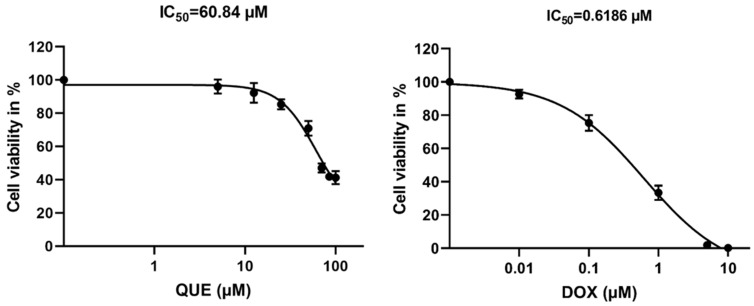
Dose–response curves for QUE and DOX monotherapies in MM28 cells following 72 h exposure. The curves were generated based on the MTT assay results, and the IC_50_ values were calculated using nonlinear regression analysis. Percent change represents the reduction in cell viability relative to the control group. Black spots represent individual data points, and the lines indicate the fitted dose–response model.

In the case of the MM28 cell line, after 72 h incubation, the IC_50_ value of QUE was calculated to be approximately 60.84 μM, while for DOX, it was 0.6186 μM (Figure 2).

The calculated IC_50_ values were used to define the concentrations applied in the combination treatment groups. Based on the determined IC_50_ values of QUE, cell line-specific concentrations were applied (50 µM and 70 µM, respectively) to ensure comparable biological exposure [17,18]. Doxorubicin exhibited similar IC_50_ values in the two cell lines. Therefore, a single concentration (1 µM) [19], corresponding approximately to the IC_50_ range, was applied in mono and combination treatments. The 1 µM DOX concentration was selected because it represents a biologically active concentration commonly used in cancer cell line studies. This concentration also allowed the evaluation of treatment-induced molecular alterations without complete loss of cell viability [19,20,21]. Although the IC_50_ values of doxorubicin differed between MEL-202 and MM28 cells, both remained within the low micromolar range. Therefore, 1 µM DOX was selected as a standardized concentration to enable the direct comparison of treatment responses and molecular alterations between the two uveal melanoma models.

**Figure 3:**
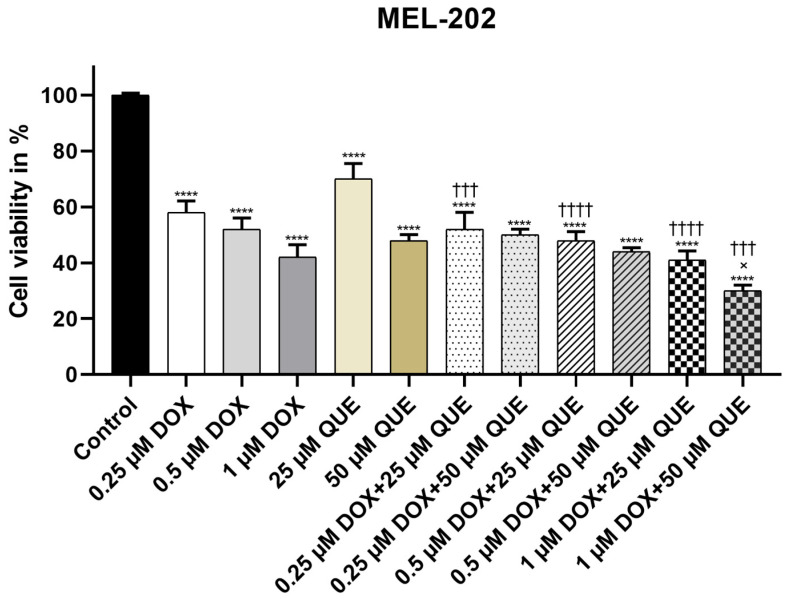
MEL-202 cells were treated with increasing concentrations of DOX (0.25, 0.5, 1 µM) and QUE (25, 50 µM) or their combinations (0.25 µM DOX+25 µM QUE, 0.25 µM DOX+50 µM QUE, 0.5 µM DOX+25 µM QUE, 0.5 µM DOX+50 µM QUE, 1 µM DOX+25 µM QUE, 1 µM DOX+50 µM QUE) for 72 h. Cell viability was measured using an MTT assay and expressed as a percentage, relative the control group. Data are expressed as the mean ± standard deviation (SD) from three independent experiments. Statistical analysis was performed using one-way ANOVA, followed by Tukey’s multiple comparison test (**** *p* < 0.0001 vs. control group, × *p* < 0.05 vs. DOX monotherapy at corresponding concentration, ††† *p* < 0.001, †††† *p* < 0.0001 vs. QUE monotherapy at corresponding concentration).

In MEL-202 cells, only one of the combination treatment groups (1 µM DOX+50 µM QUE) showed significantly lower cell viability compared to corresponding monotherapies of DOX (× *p* = 0.0170) and QUE (††† *p* = 0.0001) (Figure 3).

**Figure 4:**
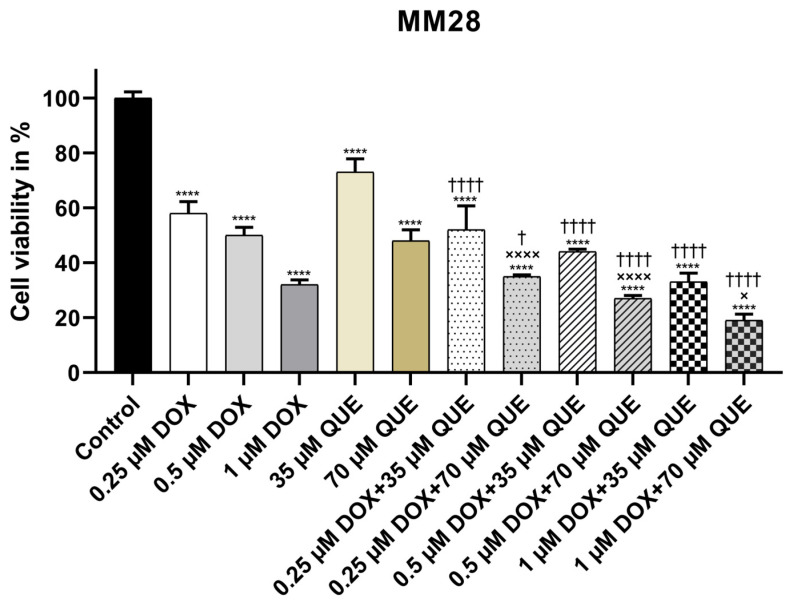
MM28 cells were treated with increasing concentrations of DOX (0.25, 0.5, 1 µM) and QUE (35, 70 µM) or their combinations (0.25 µM DOX+35 µM QUE, 0.25 µM DOX+70 µM QUE, 0.5 µM DOX+35 µM QUE, 0.5 µM DOX+70 µM QUE, 1 µM DOX+35 µM QUE, 1 µM DOX+70 µM QUE) for 72 h. Cell viability was measured using the MTT assay and expressed as a percentage, relative to the control group. Data are expressed as the mean ± standard deviation (SD) from three independent experiments. Statistical analysis was performed using one-way ANOVA, followed by Tukey’s multiple comparison test (**** *p* < 0.0001 vs. control group, × *p* < 0.05, ×××× *p* < 0.0001 vs. DOX monotherapy at corresponding concentration, † *p* < 0.05, †††† *p* < 0.0001 vs. QUE monotherapy at corresponding concentration).

In MM28 cells, combination treatment groups (0.25 µM DOX+70 µM QUE, 0.5 µM DOX+70 µM QUE and 1 µM DOX+70 µM QUE) showed significantly lower cell viability compared to both their respective DOX and QUE monotherapy. However, the lowest cell viability resulted in the 1 µM DOX+70 µM QUE treatment (~19%) vs. control group (Figure 4).

### 3.2. QUE Mono and Combination Therapy Decreased the Protein Expressions of Markers Related to the PI3K/AKT Pathway

**Figure 5:**
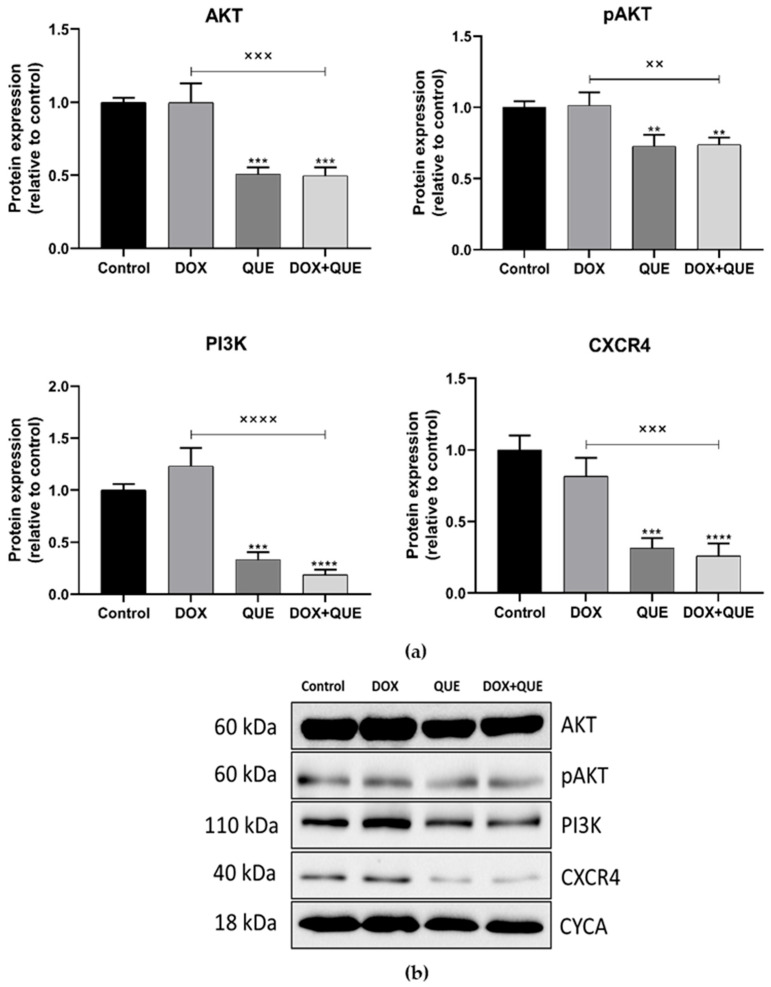
Western blot analysis of AKT, pAKT, PI3K and CXCR4 protein expression in MEL-202 cells following treatment with 1 µM DOX, 50 µM QUE or their combination for 72 h. The expression levels were normalized to untreated controls (control expression = 1). (**a**) Relative protein expression levels. (**b**) Representative Western blot images. Data are presented as mean ± SD from three independent experiments. Statistical significance was determined by one-way ANOVA followed by Tukey’s multiple comparison test (** *p* < 0.01, *** *p* < 0.001, **** *p* < 0.0001 vs. control, ×× *p* < 0.01, ××× *p* < 0.001, ×××× *p* < 0.0001 vs. DOX).

In the following experiments, we studied the effects of 1 µM DOX, 50 µM and 70 µM QUE or their combination treatment for 72 h on proteins involved in the PI3K/AKT pathway, focusing on PI3K, AKT and phosphorylated-AKT (pAKT). According to Western blot analysis with MEL-202 cells, the expression levels of AKT and pAKT decreased significantly in 50 µM QUE monotherapy (*** *p* = 0.0002, ** *p* = 0.0059, respectively) and combination therapy (*** *p* = 0.0002, ** *p* = 0.0076); however, the 1 µM DOX monotherapy showed no significant change in either case (Figure 5). Notably, the combination therapy caused a significant change when compared to the 1 µM DOX monotherapy in AKT (××× *p* = 0.0002) and pAKT (×× *p* = 0.0057) (Figure 5).

Moreover, PI3K showed a significant reduction in 50 µM QUE monotherapy (*** *p* = 0.0002); although the combined therapy caused a more prominent reduction (**** *p* < 0.0001) in the protein expression, 1 µM DOX monotherapy caused no significant change relative to control group. Accordingly, compared to 1 µM DOX, combination therapy showed a significant decrease (×××× *p* < 0.0001) (Figure 5).

The CXCR4 protein showed a significant reduction in 50 µM QUE monotherapy (*** *p* = 0.0001) and combination therapy (**** *p* < 0.0001) vs. control (Figure 5). Combination treatment showed a significant decrease (××× *p* = 0.0006) compared to 1 µM DOX treatment.

**Figure 6:**
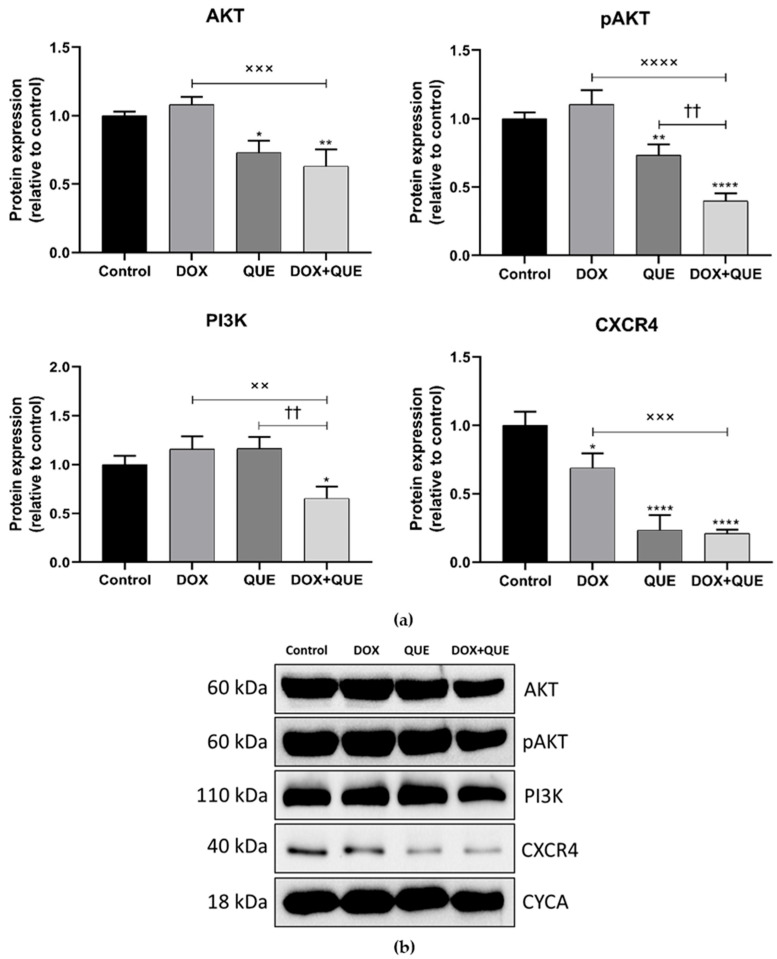
Western blot analysis of AKT, pAKT, PI3K and CXCR4 protein expression in MM28 cells following treatment with 1 µM DOX, 70 µM QUE or their combination for 72 h. The expression levels were normalized to untreated controls (control expression = 1). (**a**) Relative protein expression levels. (**b**) Representative Western blot images. Data are presented as mean ± SD from three independent experiments. Statistical significance was determined by one-way ANOVA followed by Tukey’s multiple comparison test (* *p* < 0.05, ** *p* < 0.01, **** *p* < 0.0001 vs. control, ×× *p* < 0.01, ××× *p* < 0.001, ×××× *p* < 0.0001 vs. DOX, †† *p* < 0.01 vs. QUE).

In MM28 cells, the protein expression levels of AKT and pAKT were decreased significantly (* *p* = 0.0156, ** *p* = 0.0100) by 70 µM QUE monotherapy relative to the control group (Figure 6). Combination therapy showed a greater reduction (** *p* = 0.0024, **** *p* = 0.0001, respectively) in both markers. Between 1 µM DOX monotherapy and combination therapy, a significant reduction was noticeable (××× *p* = 0.0007, ×××× *p* < 0.0001); however, only pAKT showed a significant reduction between 70 µM QUE monotherapy and combination therapy (†† *p* = 0.0026) (Figure 6).

The PI3K expression resulted in a significant reduction only with the combination therapy (* *p* = 0.0263) and demonstrated a significant decrease compared to 1 µM DOX monotherapy (×× *p* = 0.0031) and 70 µM QUE monotherapy (†† *p* = 0.0029) (Figure 6).

Western blot results showed a significant reduction in CXCR4 expression with 1 µM DOX monotherapy (* *p* = 0.0143), 70 µM QUE monotherapy (*** *p* < 0.0001) and combination therapy (**** *p* < 0.0001) relative to the control group (Figure 6). Combination treatment showed a significant decrease (××× *p* = 0.0010) versus 1 µM DOX treatment.

### 3.3. Treatment-Induced Suppression of NF-κB and Activation of p53 Proteins

**Figure 7:**
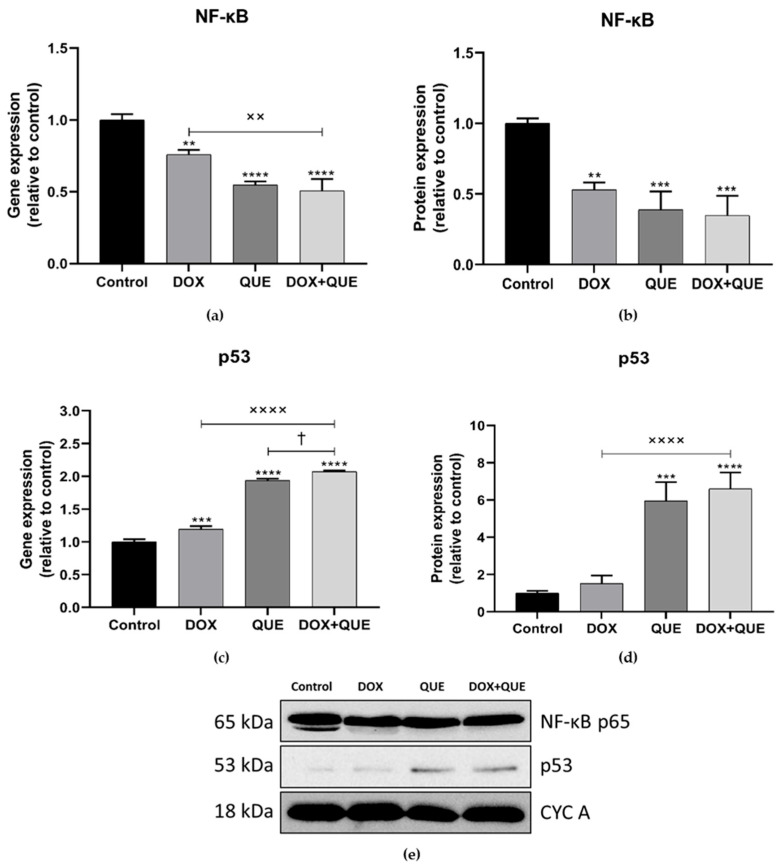
qPCR and Western blot analysis of NF-κB and p53 expression in MEL-202 cells following treatment with 1 µM DOX, 50 µM QUE or their combination for 72 h. The expression levels were normalized to untreated controls (control expression = 1). (**a**,**c**) Relative gene expression levels. (**b**,**d**) Relative protein expression levels. (**e**) Representative Western blot images. Data are presented as mean ± SD from three independent experiments. Statistical significance was determined by one-way ANOVA followed by Tukey’s multiple comparison test (** *p* < 0.01, *** *p* < 0.001, **** *p* < 0.0001 vs. control, ×× *p* < 0.01, ×××× *p* < 0.0001 vs. DOX and † *p* < 0.05 vs. QUE).

Our results demonstrated that NF-κB showed a significant reduction both in gene and protein expression after 1 µM DOX, 50 µM QUE and their combination treatment in MEL-202 cells. However, the combination therapy showed a significant reduction only when compared to 1 µM DOX monotherapy (×× *p* = 0.0013) in gene expression (Figure 7).

Expression of the p53 gene and the corresponding protein showed a similar increase after 50 µM QUE monotherapy and combination treatments. The combination therapy resulted in a significant increase in gene expression vs. 1 µM DOX monotherapy (×××× *p* < 0.0001) and 50 µM QUE monotherapy († *p* = 0.0104) (Figure 7).

**Figure 8:**
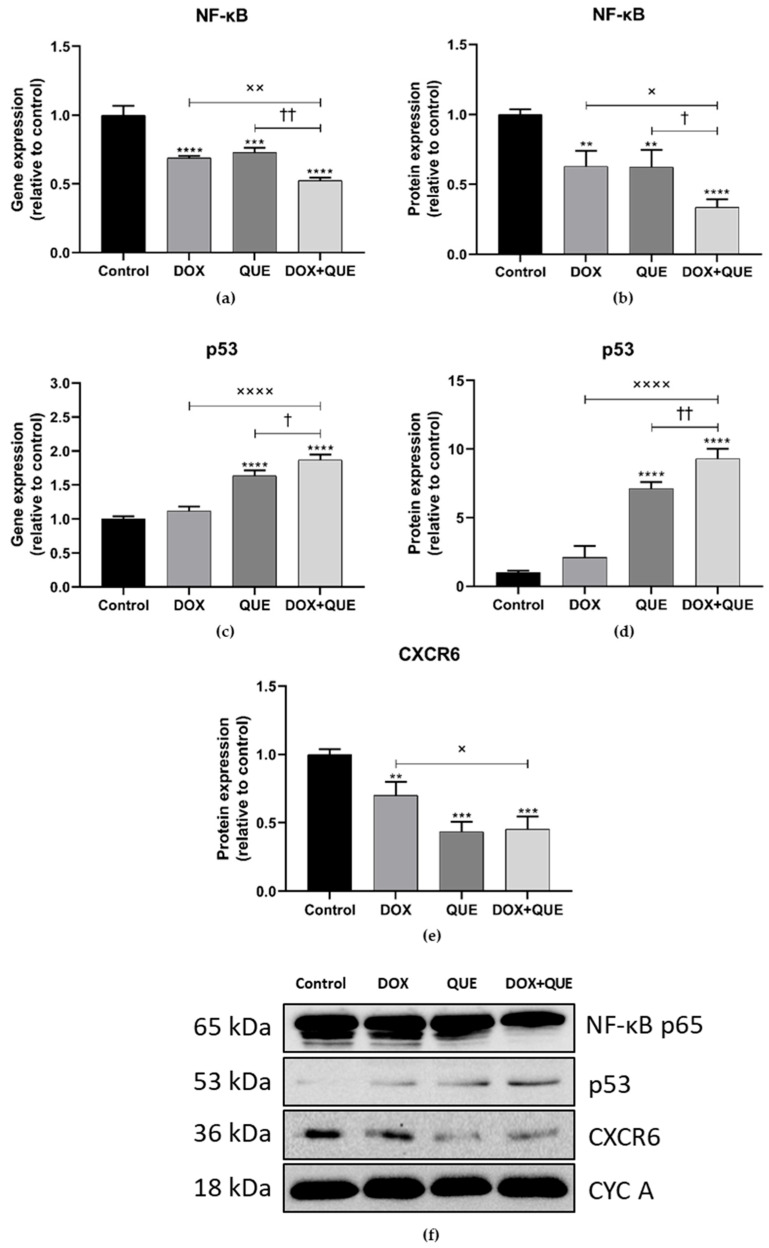
qPCR and Western blot analysis of NF-κB, p53 and CXCR6 expression in MM28 cells following treatment with 1 µM DOX, 70 µM QUE or their combination for 72 h. The expression levels were normalized to untreated controls (control expression = 1). (**a**,**c**) Relative gene expression levels. (**b**,**d**,**e**) Relative protein expression levels. (**f**) Representative Western blot images. Data are presented as mean ± SD from three independent experiments. Statistical significance was determined by one-way ANOVA followed by Tukey’s multiple comparison test (** *p* < 0.01, *** *p* < 0.001, **** *p* < 0.0001 vs. control, × *p* < 0.05, ×× *p* < 0.01, ×××× *p* < 0.0001 vs. DOX, † *p* < 0.05, †† *p* < 0.01 vs. QUE).

The effects of 1 µM DOX, 70 µM QUE, and their combination on NF-κB and p53 expression were evaluated at both gene and protein levels. In MM28 cells, the mRNA and protein expression of NF-κB was significantly reduced following all treatments relative to the control group. At the mRNA level, both 1 µM DOX and 70 µM QUE monotherapies and the combination therapy induced a marked decrease (*** *p* = 0.0002, **** *p* < 0.0001) (Figure 8). Importantly, the combination treatment decreased mRNA expression of NF-κB compared to 1 µM DOX (×× *p* = 0.0053) and 70 µM QUE (†† *p* = 0.0013) monotherapies, indicating a more pronounced inhibitory effect. A similar trend was observed at the protein level, where 1 µM DOX and 70 µM QUE significantly reduced NF-κB protein expression (** *p* < 0.01), and the combination treatment led to a further significant reduction (*p* < 0.0001), with statistically significant differences vs. both single treatments (*p* < 0.05) (Figure 8).

In contrast, p53 expression showed an opposite pattern on both mRNS and protein levels. At the gene level, 70 µM QUE significantly increased p53 expression (**** *p* < 0.0001), while 1 µM DOX alone had only a modest effect. The combination treatment resulted in a further significant upregulation relative to the control (**** *p* < 0.0001) and 1 µM DOX (×××× *p* < 0.0001), and was also significantly higher than 70 µM QUE alone († *p* = 0.0124) (Figure 8). At the protein level, a strong induction of p53 was observed following 70 µM QUE treatment (**** *p* < 0.0001), which was further increased in the combination group, when compared to 1 µM DOX (**** *p* < 0.0001) and 70 µM QUE (†† *p* = 0.0090) alone.

In case of CXCR6, 1 µM DOX treatment caused a significant decrease (** *p* = 0.0074); furthermore, 70 µM QUE monotherapy (*** *p* = 0.0001) and combination therapy (*** *p* = 0.0001) significantly reduced the protein expression. Combination treatment showed a significant decrease (× *p* = 0.0216) versus 1 µM DOX treatment (Figure 8).

Overall, these results suggest that the combined 1 µM DOX+70 µM QUE treatment was associated with greater suppression of mRNA and protein levels of NF-κB and increased p53 expression compared to either monotherapy (Figure 8).

### 3.4. AKT1 Downregulation in MEL-202 but Not in MM28 Cells

**Figure 9:**
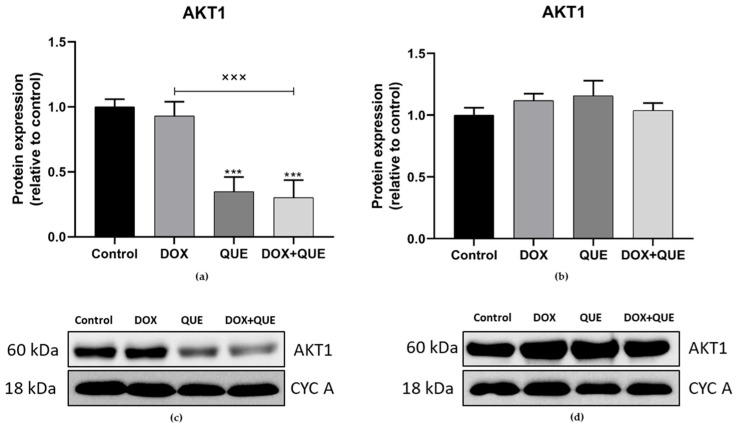
Western blot analysis of AKT1 protein expression in MEL-202 and MM28 cells following treatment with 1 µM DOX, 50 µM or 70 µM QUE, or their combination, for 72 h. The expression levels were normalized to untreated controls (control expression = 1). (**a**) Relative AKT1 protein expression levels in MEL-202. (**b**) Relative AKT1 protein expression levels in MM28. (**c**,**d**) Representative Western blot images. Data are presented as mean ± SD from three independent experiments. Statistical significance was determined by one-way ANOVA followed by Tukey’s multiple comparison test (*** *p* < 0.001 vs. control, ××× *p* < 0.001 vs. DOX).

According to Western blot results, in MEL-202 cells, the AKT1 protein showed a significant reduction in 50 µM QUE monotherapy (*** *p* = 0.0004) and combination therapy (*** *p* = 0.0002) vs. control (Figure 9a). Combination treatment showed a significant decrease (××× *p* = 0.0005) compared to 1 µM DOX monotherapy. Interestingly, AKT1 showed no significant change with the therapies in MM28 cells (Figure 9b).

### 3.5. Different Modulations of Matrix Metalloproteinase (MMP) Proteins Following Treatments

**Figure 10:**
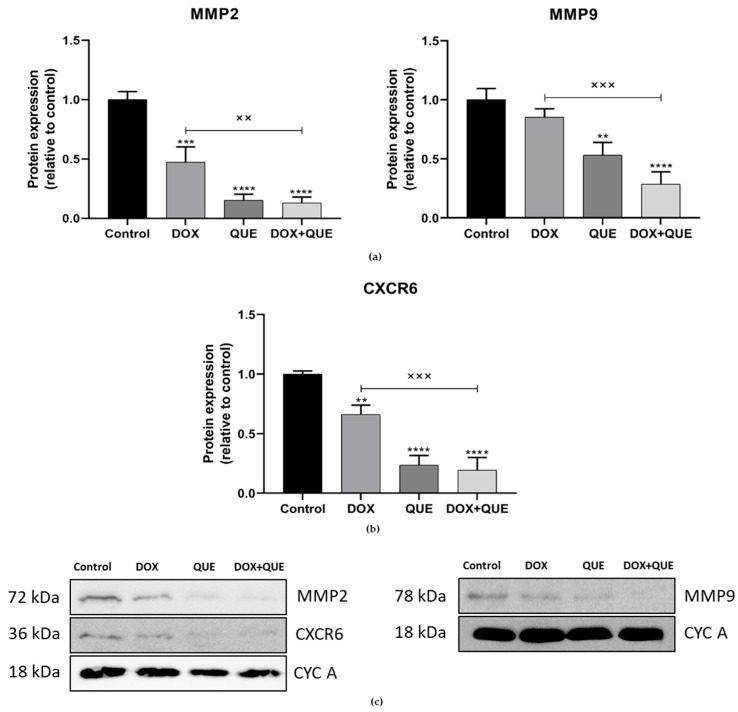
Western blot analysis of MMP2, MMP9 and CXCR6 protein expression in MEL-202 cells following treatment with 1 µM DOX, 50 µM QUE or their combination for 72 h. The expression levels were normalized to untreated controls (control expression = 1). (**a**,**b**) Relative protein expression levels. (**c**) Representative Western blot images. Data are presented as mean ± SD from three independent experiments. Statistical significance was determined by one-way ANOVA followed by Tukey’s multiple comparison test (** *p* < 0.01, *** *p* < 0.001, **** *p* < 0.0001 vs. control, ×× *p* < 0.01, ××× *p* < 0.001 vs. DOX).

In MEL-202 cells, the effects of 1 µM DOX, 50 µM QUE and their combination on MMP2 and MMP9 expression were evaluated at the protein level. MMP2 expression was significantly reduced following 1 µM DOX (*** *p* = 0.0002), 50 µM QUE (**** *p* < 0.0001) and combination therapy (**** *p* < 0.0001) relative to the control group (Figure 10). Combination therapy caused a significant reduction (×× *p* = 0.0038) in the MMP2 protein level compared to 1 µM DOX.

Similarly, the MMP9 protein level was significantly decreased by 50 µM QUE (** *p* = 0.0014) and combination therapy (**** *p* < 0.0001) vs. control. Combination therapy showed a significant reduction (××× *p* = 0.0004) in protein expression than observed in 1 µM DOX-treated cells (Figure 10).

In the case of CXCR6, 1 µM DOX treatment caused a significant decrease (** *p* = 0.0033); furthermore, 50 µM QUE monotherapy (**** *p* < 0.0001) and combination therapy (**** *p* < 0.0001) reduced the protein expression significantly. Combination treatment showed a significant decrease (××× *p* = 0.0004) compared to 1 µM DOX treatment (Figure 10b).

**Figure 11:**
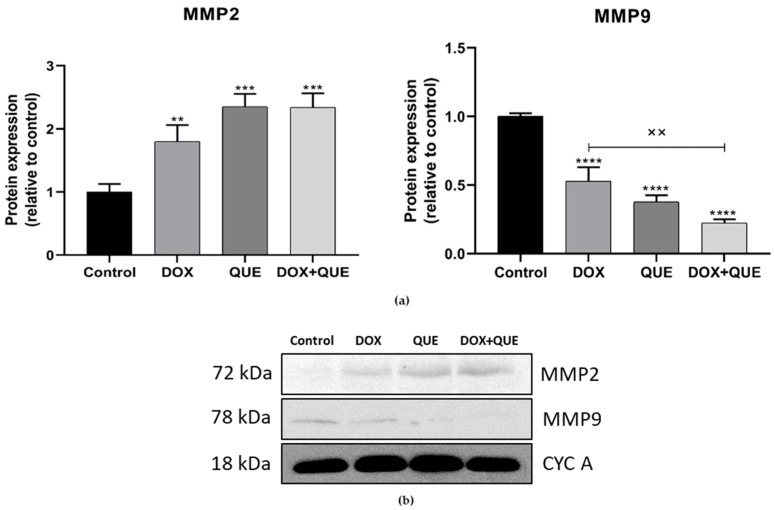
Western blot analysis of MMP2 and MMP9 protein expression in MM28 cells following treatment with 1 µM DOX, 70 µM QUE or their combination for 72 h. The expression levels were normalized to untreated controls (control expression = 1). (**a**) Relative protein expression levels. (**b**) Representative Western blot images. Data are presented as mean ± SD from three independent experiments. Statistical significance was determined by one-way ANOVA followed by Tukey’s multiple comparison test (** *p* < 0.01, *** *p* < 0.001, **** *p* < 0.0001 vs. control, ×× *p* < 0.01 vs. DOX).

In MM28 cells, MMP2 expression was significantly increased following 1 µM DOX (** *p* = 0.0069), 70 µM QUE (*** *p* = 0.0002) and combination therapy (*** *p* = 0.0002) relative to the control group (Figure 11).

Interestingly, protein expression of MMP9 was significantly decreased (**** *p* < 0.0001) by any treatments used. Combination therapy showed a significant reduction (×× *p* = 0.0011) compared to 1 µM DOX monotherapy (Figure 11).

### 3.6. Reduction in Post-Treatment Invasive Capacity in UM Cell Lines

**Figure 12:**
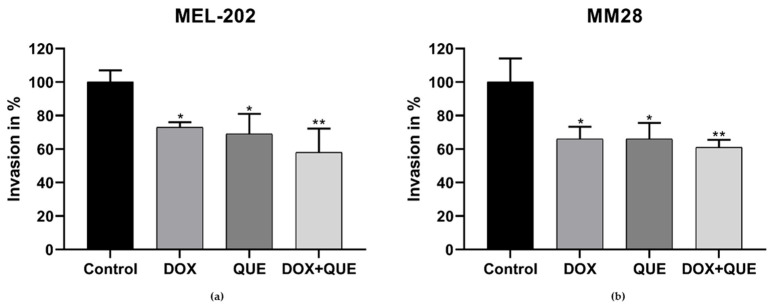
Effect of DOX, QUE and their combination on the post-treatment invasion capacity of MEL-202 and MM28 cells following 72 h treatment. Cells were treated with 1 µM DOX, 50 µM QUE (MEL-202) or 70 µM QUE (MM28), alone or in combination, prior to invasion assay analysis. (**a**) Relative invasion of MEL-202 cells. (**b**) Relative invasion of MM28 cells. Data are presented as mean ± SD from three independent experiments. Statistical significance was determined by one-way ANOVA followed by Tukey’s multiple comparison test (* *p* < 0.05, ** *p* < 0.01 vs. control).

The invasive capacity of MEL-202 and MM28 cells was evaluated using a QCM^TM^ 96-well invasion assay, and the results were expressed as a percentage relative to the control group. Treatment of MEL-202 with 1 µM DOX or 50 µM QUE alone significantly reduced (* *p* = 0.0457 and * *p* = 0.0234, respectively) the post-treatment invasive potential of MEL-202 cells vs. control. Notably, the combination therapy of 1 µM DOX+50 µM QUE resulted in a more pronounced inhibition of cell invasion than monotherapies (** *p* = 0.0042) relative to the control group (Figure 12a).

In MM28 cells, treatment with either 1 µM DOX or 70 µM QUE alone significantly decreased post-treatment invasive potential compared with the control (* *p* = 0.0104 in both cases). Similarly to MEL-202, the 1 µM DOX+70 µM QUE combination led to a more pronounced inhibition of invasion (** *p* = 0.0047) relative to the control group (Figure 12b).

### 3.7. Cell Cycle Analysis by Flow Cytometry

**Figure 13:**
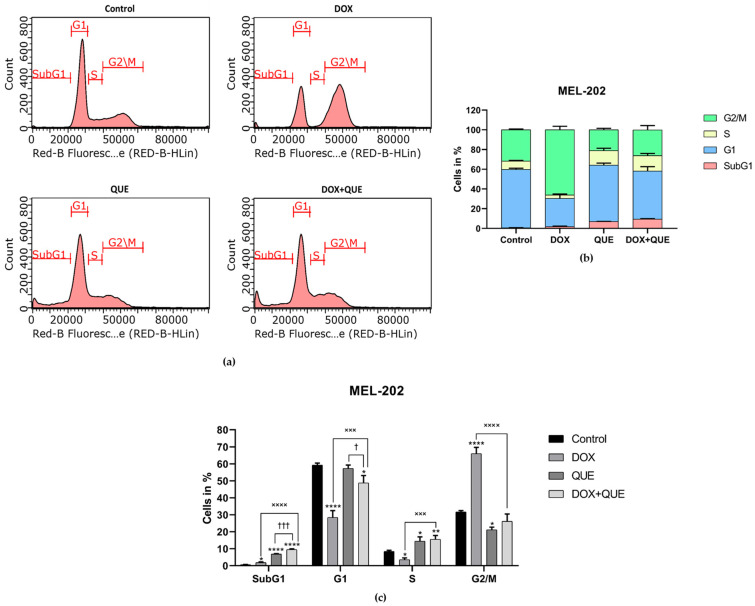
Flow cytometry analysis of cell cycle distribution in MEL-202 cells following treatment with 1 µM DOX, 50 µM QUE or their combination for 72 h. (**a**) Representative histograms. (**b**,**c**) Percentage distribution of cells in different cell cycle phases. Data are presented as mean ± SD from three independent experiments. Statistical significance was determined by one-way ANOVA followed by Tukey’s multiple comparison test (* *p* < 0.05, ** *p* < 0.01, **** *p* < 0.0001 vs. control, ××× *p* < 0.001, ×××× *p* < 0.0001 vs. DOX and † *p*< 0.05, ††† *p* < 0.001 vs. QUE).

The cell cycle distribution of MEL-202 cells was analyzed following 72 h treatment with 1 µM doxorubicin, 50 µM quercetin, and their combination (Figure 13).

In control cells, the majority of the population was detected in the G1 phase (~59.35%), with smaller fractions in the S phase (~8.45%) and G2/M phase (~31.67%), while the SubG1 population remained minimal, indicating low basal apoptotic DNA fragmentation.

Treatment of 1 µM DOX resulted in a complete redistribution of the cell cycle, defined by a large accumulation of cells in the G2/M phase (~66.11%), accompanied by a major reduction in the G1 population (~28.45%). The SubG1 fraction remained low, suggesting that DOX primarily induced G2/M arrest rather than extensive DNA fragmentation under these conditions (Figure 13).

In contrast, 50 µM QUE treatment increased the SubG1 population (~6.91%) compared to the control, accompanied by a moderate decrease in G1 and slight alterations in S and G2/M phases. These findings may indicate that QUE promoted DNA fragmentation and moderately affected cell cycle progression (Figure 13).

Combination therapy led to the highest SubG1 proportion (~9.48%), together with a reduction in G1 and a moderate G2/M accumulation.

Altogether, these data suggest that DOX dominantly induces G2/M arrest, whereas QUE and the combination treatment are associated with increased DNA fragmentation in MEL-202 cells (Figure 13).

**Figure 14:**
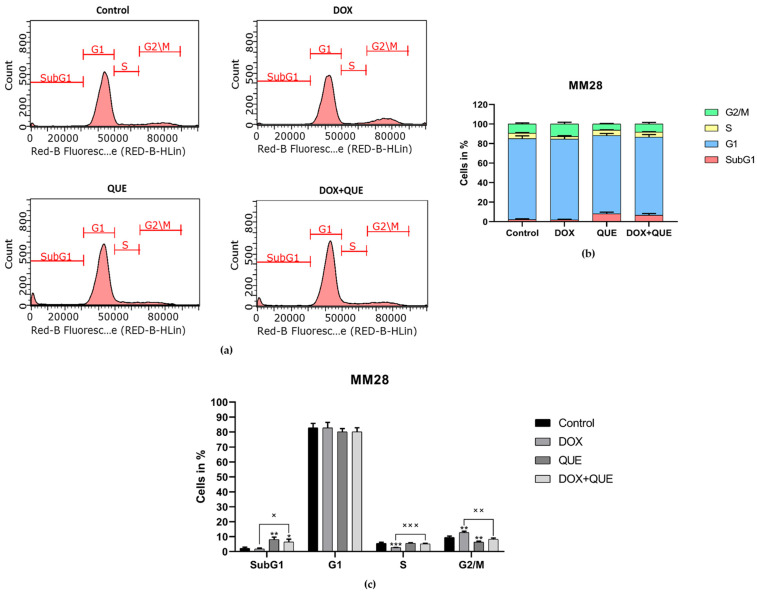
Flow cytometry analysis of cell cycle distribution in MM28 cells following treatment with 1 µM DOX, 70 µM QUE or their combination for 72 h. (**a**) Representative histograms. (**b**,**c**) Percentage distribution of cells in different cell cycle phases. Data are presented as mean ± SD from three independent experiments. Statistical significance was determined by one-way ANOVA followed by Tukey’s multiple comparison test (* *p* < 0.05, ** *p* < 0.01, *** *p* < 0.001 vs. control and × *p* < 0.05, ×× *p* < 0.01, ××× *p* < 0.001 vs. DOX).

The cell cycle distribution of MM28 cells after treatment with 1 µM DOX, 70 µM QUE, and their combination (DOX+QUE) was analyzed by flow cytometry (Figure 14). In control cells, the majority of the population was in the G1 phase, approximately 82.85%, while smaller fractions were present in the S phase (~5.51%) and G2/M phase (~9.46%). The rate of SubG1 cells, representing cells with fragmented DNA, remained low (~2.18%).

Treatment with 1 µM DOX alone did not considerably change the overall cell cycle distribution, as seen in MEL-202 cells. Most of the cells remained in the G1 phase, while moderate changes were observed in the S (~2.70%) and G2/M (~12.75%) fractions. Similarly, the SubG1 population remained low (~1.81%), indicating limited induction of DNA fragmentation.

In contrast, 70 µM QUE treatment resulted in a significant increase in the SubG1 population, approximately 8.09%, consistent with increased apoptotic cell death. This increase was accompanied by a slight reduction in the G1 fraction (~80.07%), while the ratio of the S phase cells remained unchanged (~5.55%); however, the G2/M phase cells were reduced to approximately 6.30% by the treatment (Figure 14).

The combined DOX+QUE treatment also elevated the SubG1 fraction (~6.50%) compared to the control and DOX-treated samples, although the increase was slightly lower than that observed with QUE alone. Overall, the majority of cells across all treatment groups remained in the G1 phase, indicating that neither monotherapy nor the combination treatment caused a pronounced arrest in a specific cell cycle phase, but QUE-containing treatments were associated with an increased proportion of SubG1 cells (Figure 14).

### 3.8. Quercetin-Driven Cell Death in UM Cells

**Figure 15:**
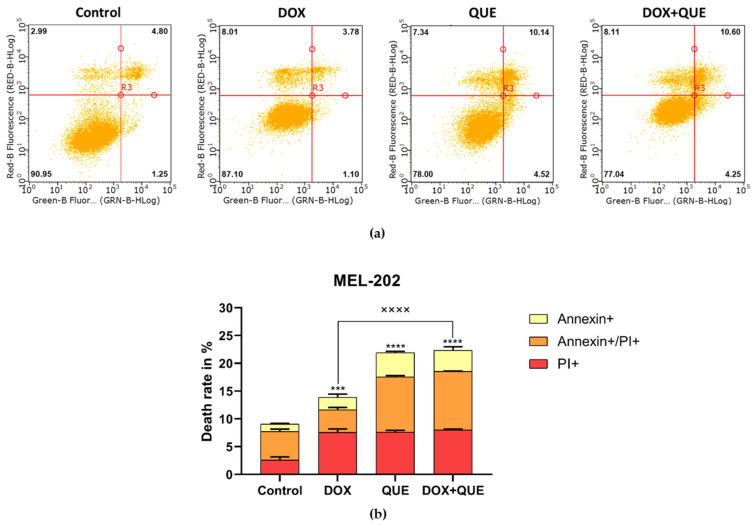
Flow cytometry analysis using Annexin V/PI-staining. (**a**) Representative dot plots of MEL-202 cells. Cells were treated with 1 μM DOX, 50 μM quercetin, or their combination for 72 h and then harvested for analysis. (**b**) Bar graph represents the death rate in percentage in MEL-202 cells caused by the therapies. Data are presented as the mean ± S.D from three independent experiments. One-way ANOVA was used for statistical analysis followed by Tukey’s multiple comparison test (*** *p* < 0.001, **** *p* < 0.0001 vs. control, ×××× *p* < 0.0001 vs. DOX).

Flow cytometry based on Annexin V-fluorescein isothiocyanate/propidium iodide (Annexin-V-FITC/PI) double staining analysis was performed to investigate the influence of DOX and QUE monotherapy or their combination. The applied treatments affected cell death in MEL-202 cells. In control samples, the majority of cells remained viable, while only a small fraction displayed apoptotic or necrotic features. The 1 µM DOX treatment moderately increased the proportion of necrotic cells (PI+) compared to the control group (Figure 15). In contrast, 50 µM QUE treatment resulted in a more pronounced elevation in apoptotic cell populations, characterized by an increase in both early (Annexin+) and late apoptotic (Annexin+/PI+) fractions. The cell death in DOX+QUE therapy was similar to that observed with QUE monotherapy. Overall, these results indicate that 50 µM QUE might induce apoptotic cell death in MEL-202 cells, while the combination with 1 µM DOX showed similar results. This is suggesting that quercetin may have been the primary contributor to cell death induction, while additional effects of the combination therapy were more marked in modulation of certain PI3K/AKT pathway-related proteins and post-treatment invasion. The observed increase in apoptotic cell populations after QUE-containing treatments occurred together with reduced AKT and pAKT expression levels, suggesting that PI3K/AKT-related signaling may be involved in the cellular response to the treatments (Figure 15).

**Figure 16:**
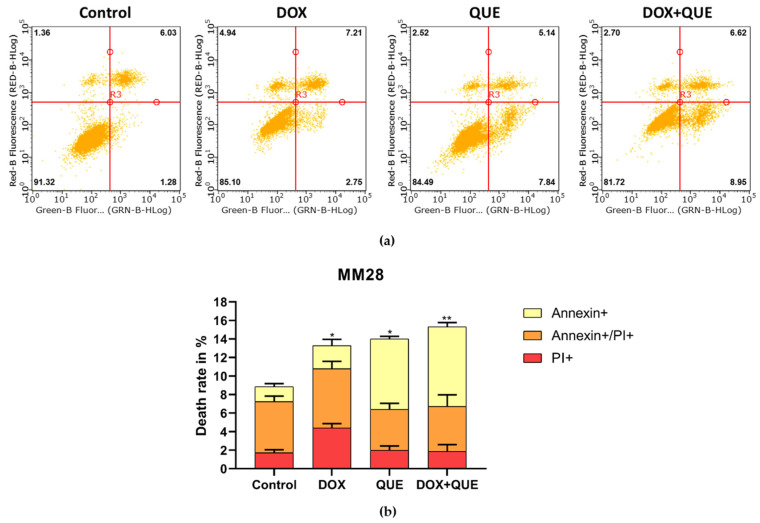
Flow cytometry analysis using Annexin V/PI-staining. (**a**) Representative dot plots of MM28 cells. Cells were treated with 1 μM DOX, 70 μM quercetin, or their combination for 72 h and then harvested for analysis. The figure represents the dot plots of treatments. (**b**) Bar graph represents the percentage of death rate. Data are presented as the mean ± SD from three independent experiments. One-way ANOVA was used for statistical analysis followed by Tukey’s multiple comparison test (* *p* < 0.05, ** *p* < 0.01 vs. control).

The Annexin V/PI assay performed on MM28 cells revealed an increase in overall cell death following all treatments relative to the control. Both 1 µM DOX and 70 µM QUE monotherapies induced a moderate elevation in apoptotic cell populations, as indicated by increased Annexin-positive fractions. The combination treatment (DOX+QUE) resulted in the highest level of total cell death (Figure 16).

More specifically, 1 µM DOX treatment led to a marked increase in both necrotic (PI+) and late apoptotic (Annexin+/PI+) populations, accompanied by a moderate rise in early apoptotic (Annexin+/PI-) cells. The 70 µM QUE treatment also elevated apoptotic cell fractions, although to a slightly lesser extent in the late apoptotic population (Figure 16). Notably, the combination treatment further increased the death rate in MM28 cells, suggesting a greater effect compared to monotherapies. Overall, these results may indicate that both agents are capable of inducing cell death in MM28 cells, with the combination treatment resulted in a greater increase (Figure 16). Similarly to MEL-202, in MM28 cells, the apoptotic response observed following QUE-containing treatments was accompanied by reduced AKT and pAKT expression levels, which may suggest that PI3K/AKT-related signaling can be involved in treatment-associated effects.

## 4. Discussion

Human uveal melanoma is a rare type of cancer, yet it is the most common primary intraocular malignancy in adults. In most cases, it originates from the choroid (90%), the ciliary body (6%), and the iris (4%) and carries a poor prognosis. Despite years of research, the treatment of UM remains in need of improvement, especially the prevention and treatment of metastatic spread [22]. Doxorubicin is a well-known anticancer agent, but its clinical use is limited by its toxicity to normal tissues, especially the heart and liver. In addition, its therapeutic use is often the cause of multidrug resistance in cancer cells. Interestingly, certain flavonoids, including quercetin, have been shown to enhance the antitumor effect of well-known chemotherapeutic agents when used in combination [23,24]. A previous study suggests that quercetin may inhibit uveal melanoma cell growth by interfering glucose uptake and metabolism [25]. Thus, we aimed to investigate its effect on primary and metastatic UM cell lines alone, or in a combination with a well-known chemotherapeutic agent, doxorubicin, focusing on cell viability, invasion, cell cycle progression, and key signaling pathways associated with tumor progression and metastasis.

Our results demonstrate that after 72 h, both agents reduced cell viability in a dose-dependent manner, while their combination led to a greater inhibitory effect. According to our results, the IC_50_ of quercetin was approximately 39.55 µM, while the IC_50_ of DOX was approximately 0.8737 µM on the MEL-202 cell line. Accordingly, in MM28 cells, we observed a higher IC_50_ of QUE, approximately 60.84 µM, with a similar IC_50_ of DOX, approximately 0.6186 µM. The IC_50_ values were used to define the concentration ranges applied in combination analyses. In the following experiments, we used 1 µM DOX for both cell lines, and 50 and 70 µM of QUE, respectively. Doxorubicin has variable cytotoxicity among various types of cancer cell lines, with IC_50_ values ranging from nanomolar to micromolar concentrations [26]. The IC_50_ for QUE has a wide range among various cancer cell lines [7,27], but it is important to mention that similarly to our results, another study stated an IC_50_ value for QUE in UM cell line 92.1 (44.05 µM) [25].

Based on previous reports, QUE can be associated with an anticancer effect by modulating the PI3K/AKT pathway [25,27]. In our study, using MEL-202 cells, the applied concentration of 1 µM DOX caused no significant change in the expression of AKT, pAKT and PI3K. On the contrary, 50 µM QUE significantly inhibited the expression of these markers relative to the control group. In the case of PI3K, the combination therapy caused a more prominent reduction in the protein expression vs. control and DOX group. The results in MM28 cells indicated a marked modulation of the PI3K/AKT-related proteins in response to the different treatments. While DOX alone induced a nonsignificant increase in total AKT and pAKT protein levels, QUE treatment caused a significant reduction, particularly in phosphorylated AKT. Notably, the combined DOX + QUE treatment resulted in the greatest decrease in pAKT and PI3K expression among the examined treatment groups. Although pAKT expression was evaluated in the present study, interpretation of AKT activity should also consider total AKT expression, as the pAKT/AKT ratio generally functions as a more direct indicator of AKT activation status.

It is well-known that the PI3K/AKT pathway regulates the expression of p53 and NF-κB. These markers have an important role in cell survival, proliferation and apoptosis [28,29]. A former study stated that QUE has a capacity to suppress cancer progression through the upregulation of p53 and the inhibition of NF-κB signaling, highlighting its potential as a promising agent for anticancer therapy [30].

We observed similar results in the expression changes in p53 and NF-κB genes and corresponding proteins, which support the involvement of key survival and apoptotic pathways in the response to the applied treatments. Both gene and protein expression analyses showed a significant downregulation of NF-κB following QUE treatment, also observed in the DOX+QUE combination group. Although DOX alone also reduced NF-κB levels, the effect was weaker compared to QUE-containing treatments, suggesting that QUE plays a dominant role in inhibiting this pro-survival signaling pathway. In contrast, p53 expression showed a consistent upregulation at both the mRNA and protein levels, particularly in response to QUE and the combined treatment relative to the untreated control. The strongest increase was observed in the DOX + QUE group, which may be associated with increased activation of p53-mediated pathways.

The results with the MM28 cell line showed a modulation of key regulators of cell survival in response to the treatments. NF-κB expression was significantly reduced at both gene and protein levels following any treatments used, with the major decrease observed in the DOX+QUE combination group. While DOX and QUE alone also suppressed NF-κB, the combination therapy resulted in a more pronounced reduction in this cell line.

In parallel, p53 expression showed a marked increase, especially in response to QUE and even more significantly in the combined treatment. Although DOX alone induced only a modest increase, the DOX+QUE group demonstrated the highest p53 expression at both mRNA and protein levels, indicating a strong activation of p53-dependent signaling. These results are corresponding with a similar study in liver cancer cells [23].

CXCR4 has a key role in tumor progression, migration and invasion. CXCR4 overexpression usually presents in patients with a poor prognosis and metastases [31]. Several studies stated that another chemokine receptor, CXCR6, has a role in cancer cell invasion, DNA repair and chemoresistance [32,33,34]. Quercetin had been shown to downregulate CXCR4 expression in cancer stem cells, causing reduced migratory and invasive potential of tumor cells [35]. The investigated UM cell lines showed a significant downregulation of both CXCR4 and CXCR6 protein expression in response to quercetin-containing treatments. While DOX alone induced a moderate decrease in the levels of these chemokine receptors, QUE treatment resulted in a significantly stronger suppression. Importantly, the combination treatment with DOX and QUE led to the most significant reduction in CXCR4 expression in MEL-202 cells compared to the untreated control. CXCR6 showed a similar profile change with the treatments, although DOX monotherapy caused a significant change in both cell lines.

A former study stated that AKT1 is involved in the regulation of tumor cell invasive behavior in triple negative breast cancer [36]. This member of the PI3K/AKT pathway showed significant reduction only in quercetin-containing treatments in MEL-202 cells. On the contrary, in MM28 cells, there were no detectable changes in the AKT1 protein expression after the treatments. This discrepancy may be due to the cell line’s metastatic profile [37,38].

MMP2 and MMP9 play a central role in cancer progression by degrading the extracellular matrix, thereby promoting tumor cell invasion, migration, and metastasis [39,40]. Quercetin has been shown to suppress cancer cell invasion by downregulating MMP2 and MMP9 expression [41].

Our results with MEL-202 cells revealed a significant reduction in both MMP2 and MMP9 protein expression following treatment, with the most significant reduction observed in the DOX+QUE group in the case of MMP9. While DOX alone led to a moderate decrease, QUE induced a significantly greater suppression, which was increased in the combination treatment.

Based on our results, the MM28 cell line showed a different regulation of MMP family members in response to the treatments. MMP2 protein expression was significantly increased after the treatments used. In contrast, MMP9 expression was significantly reduced across all treatment groups. While the increase in MMP2 could indicate a compensatory or metastasis-related response, the downregulation of MMP9 could reflect a reduction in invasive potential. This different pattern suggests that MMP2 and MMP9 could be regulated differently in MM28 cells, which may be due to the metastatic cell line’s different response to certain treatments [42]. In addition, similar findings suggested that MMP2 and MMP9 may exhibit different regulation depending on the cell line and the treatment conditions. Furthermore, apoptosis-associated cellular stress may also induce the upregulation of certain MMPs independently of invasive behavior. Therefore, the observed increase in MMP2 expression does not necessarily contradict the overall anti-invasive effects detected in our study [43,44,45]. Overall, the significant inhibition of MMP9 in the combination group supports the view that quercetin may contribute to the anti-metastatic effects of doxorubicin [46], although the increased MMP2 protein expression requires more investigation to fully understand their biological function.

Results of the invasion assay demonstrate that the treatments significantly reduced the invasive capacity of both MEL-202 and MM28 cells. DOX and QUE monotherapies led to a moderate but significant decrease in invasion compared to the untreated control, while the combined DOX+QUE treatment produced the most pronounced inhibition in both models, similar to a former study [46]. A consistent trend was observed across both cell lines, indicating that the anti-invasive effects of the treatments are not cell line-specific. These findings are in line with the observed downregulation of metastasis-associated markers such as CXCR4, CXCR6 and MMP9, suggesting that quercetin may contribute to the anti-metastatic activity of doxorubicin. Altogether, our results show that beyond the inhibitory effect on some members of the PI3K/AKT pathway, the combination treatment may impair invasive behavior, which is a critical step in tumor progression and metastasis. Our findings are consistent with previous studies demonstrating that quercetin may have inhibitory effects in various types of cancer cells [47,48,49]. As cells were pre-treated prior to seeding into invasion chambers, the assay primarily reflected the invasive behavior of post-treatment surviving cells. However, treatment-induced reductions in metabolic activity may contribute to the decreased invasion observed.

The cell cycle analysis with MEL-202 cells showed different modifications in phase distribution following treatments. Both QUE and the DOX+QUE treatment significantly increased the number of cells in the SubG1 phase, consistent with DNA fragmentation [50]. This effect was significantly greater in the combination group. Moreover, the number of cells in the S phase and G2/M phase increased, specially following DOX treatment, which is consistent with its known mechanism of inducing cell cycle arrest [51]. Several studies report that quercetin may induce cell cycle arrest in cancer cells, likely in the G1, S, or G2/M phase depending on the cell line, concentration, and treatment duration [52,53,54].

In contrast, MM28 cells only demonstrated minor alterations compared to MEL-202, indicating a resistant model. A significant increase in the SubG1 population was observed following QUE and DOX+QUE treatments, suggesting the induction of DNA fragmentation. In contrast to the MEL-202 model, the number of cells in the G1 phase remained constant with any treatment groups. A significant decrease in the S phase resulted from the DOX treatment, while QUE and the combination showed no significant effects. In addition, DOX induced G2/M phase arrest; however, this effect was reduced in the combination group. Overall, these findings suggest that MM28 cells show reduced sensitivity to DOX or QUE-induced cell cycle arrest. The decreased redistribution of cell cycle phases could mean that this metastatic cell line may possess enhanced resistance mechanisms [55].

These results are consistent with previous reports demonstrating that quercetin may not induce significant cell cycle changes in certain cancer cell lines. Moreover, it has been shown that quercetin may reduce doxorubicin-induced cell cycle arrest depending on the cell line [56].

The Annexin V/PI staining in MEL-202 cells revealed that all treatments significantly increased the proportion of dead cells relative to the untreated control. Doxorubicin and quercetin monotherapy resulted in a significant increase in death rate vs. control. However, the combination therapy showed a significant increase compared to the DOX group only.

In contrast, MM28 cells showed a different response to the treatments used, where the overall cell death was lower than that observed in MEL-202 cells. In addition, the combination treatment showed the highest death rate relative to the control group. However, treatment with QUE and combination therapy caused a shift toward early apoptotic (Annexin+) cells. The metastatic nature of the cell line may be the cause of this enhanced resistance to apoptosis-inducing stimuli [57,58].

The distribution of Annexin V+ and PI+ populations supports these findings, as MEL-202 cells showed a marked increase in late apoptotic (Annexin+/PI+) cells following QUE and combination treatment, whereas MM28 cells showed a limited shift toward late apoptosis.

The different responses observed between MEL-202 and MM28 cells may reflect fundamental biological differences between primary and metastatic uveal melanomas. MM28 cells are derived from a metastatic lesion and exhibit BAP1 deficiency, a molecular feature associated with aggressive disease and a poor prognosis. Metastatic tumor cells may acquire adaptive mechanisms that promote survival under stressful conditions, including enhanced resistance to apoptosis and growth inhibition [1,2]. These characteristics may explain why MM28 cells showed weaker responses in several experimental endpoints, including AKT1 expression, cell cycle redistribution and late apoptosis, compared with MEL-202 cells.

These results are consistent with previous studies, where doxorubicin may induce apoptosis through DNA damage [59], while quercetin can govern apoptotic pathways [7] and sensitize cancer cells to chemotherapeutic agents [60]. However, the different responses observed between UM cell lines highlight the importance of tumor heterogeneity, especially the increased resistance of metastatic uveal melanoma cells, which may involve survival pathways such as PI3K/AKT signaling [61].

Although apoptosis was measured using Annexin V/PI staining, further studies investigating caspase activation and other molecular markers will be required to clarify the mechanisms behind these observations.

Interestingly, the significant reduction in cell viability demonstrated in the MTT assay was not fully consistent with the apoptotic cell fractions detected by Annexin V/PI staining, especially in the MM28 cell line. This discrepancy suggests that the anticancer agents used may decrease metabolic activity rather than cell viability. This may explain why the decrease in MTT activity was more pronounced than the apoptotic fractions detected by Annexin V/PI staining. As the MTT assay shows mitochondrial metabolic activity [62], decreased cell viability may indicate reduced metabolism instead of cell death alone. Another study stated that quercetin treatment may induce apoptosis accompanied by autophagy in HL-60 cells [63] and pro-apoptotic autophagy in non-small-cell lung cancer cell lines [64].

This conclusion is further supported by the cell cycle analysis, where the population of SubG1 in the combination treatment is significantly increased compared to DOX and QUE monotherapy in MEL-202 cells. Therefore, the reduced viability observed in the MTT assay may reflect a combination of apoptosis induction and impaired metabolism.

Moreover, in MM28 cells, the modest increase in late apoptotic fraction despite a significant decrease in viability may indicate the presence of resistance mechanisms, where cells may evade apoptosis but remain metabolically compromised [65]. Together, these findings could mean that the antitumor effects of the treatments may be mediated by multiple processes, including apoptosis, cell cycle arrest, and metabolic inhibition.

Our findings are in agreement with previous studies demonstrating that quercetin suppressed melanoma cell viability, induced the apoptotic death rate and inhibited invasive behavior through modulation of PI3K/AKT pathway-related proteins [66,67,68]. Similarly, earlier reports showed that doxorubicin induced apoptotic cell death and cell cycle arrest in melanoma models [69,70]. The reduced viability, increased apoptotic fractions, and altered PI3K/AKT-associated protein expression observed in the present study are therefore consistent with previously published findings about melanoma [66,67,68,69,70].

Previous studies demonstrated that simultaneous targeting of multiple signaling pathways may produce greater antitumor effects in UM cells than monotherapies alone. Khalili et al. reported that combined inhibition of MEK and PI3K signaling markedly enhanced apoptosis in GNAQ/GNA11-mutant uveal melanoma cells, whereas single-agent inhibition alone showed limited efficacy [13]. Similarly, another study demonstrated that the class III HDAC inhibitor Tenovin-6 combined with vinblastine enhanced apoptosis and suppressed migration in UM cell lines, suggesting that modulation of multiple cellular pathways may increase treatment sensitivity and reduce metastatic behavior [14]. These observations are in agreement with our results, where the DOX+QUE combination treatment was associated with greater modulation of PI3K/AKT-related proteins together with reduced post-treatment invasive capacity compared to monotherapies. These findings support the concept that co-targeting survival-related signaling pathways may improve therapeutic responses in UM.

In summary, our findings demonstrate that both doxorubicin and quercetin show significant antitumor effects in uveal melanoma cells, affecting cell viability, apoptosis, cell cycle progression, and invasive capacity. While both agents showed potential as monotherapies, the combination treatment was associated with more pronounced inhibition of post-treatment invasion capacity and modulation of certain members of the PI3K/AKT pathway. The inhibition on different markers of this pathway highlights the key point of tumor heterogeneity and suggests that metastatic uveal melanoma cells may possess resistance mechanisms, involving survival pathways such as PI3K/AKT [13,71,72]. Although alterations in AKT, pAKT and PI3K expression suggest that the PI3K/AKT signaling pathway may be involved in the cellular response to doxorubicin, quercetin and their combination, the present study did not investigate upstream or downstream regulators, such as PTEN, mTOR, BAD, BCL-2 or caspases. Therefore, these findings should be interpreted as pathway-associated changes rather than evidence of inhibition. Further studies including additional apoptotic and PI3K/AKT-related markers are required to clarify the precise molecular mechanisms involved. Importantly, quercetin showed anticancer activity across multiple assays, supporting its potential as a therapeutic agent. However, the limited additional effect of the combination in UM cells indicates that its therapeutic use may be cell line-dependent. From a translational perspective, the present findings are relevant since effective systemic treatment options for metastatic uveal melanoma remain limited. Although the current study was performed exclusively in vitro, the observed reduction in post-treatment invasive capacity together with modulation of survival-related proteins suggests that quercetin-containing combination strategies may have the potential to complement therapeutic approaches. As quercetin has been reported to promote the efficacy of several anticancer agents while exhibiting relatively low toxicity in preclinical models, its use as an adjuvant compound may represent a promising strategy for future research [7,73]. Nevertheless, further pharmacological, preclinical and in vivo studies are necessary to determine whether the observed effects can be translated into a clinically therapeutic approach. In conclusion, treatment with DOX and QUE and their combination suggest that quercetin-containing combination strategies may have potential in uveal melanoma treatment, although further molecular, pharmacological and in vivo studies are required to validate these findings and optimize therapeutic approaches.

## 5. Conclusions

Our results demonstrate that both doxorubicin and quercetin show significant antitumor effects in uveal melanoma cells, influencing members of the PI3K/AKT pathway, apoptosis, cell cycle progression, and invasive behavior. The combination treatment is associated with changes in PI3K/AKT-related protein expression and invasion inhibition. These findings highlight the importance of tumor heterogeneity in the therapeutic response and support the potential of quercetin as an adjuvant in uveal melanoma treatment. Further studies are needed to clarify the underlying molecular mechanisms and to validate these effects in vivo.

## Data Availability

The original contributions presented in this study are included in the article/Supplementary Materials. Further inquiries can be directed to the corresponding authors.

## Supplementary Materials

The following supporting information can be downloaded at https://www.mdpi.com/article/10.3390/cimb48060636/s1.

## Abbreviations

The following abbreviations are used in this manuscript:

AKT	Protein kinase B
Annexin V- FITC/PI	Annexin V-fluorescein isothiocyanate/propidium iodide
cDNA	Complementary DNA
CYC A	Cyclophilin A
MMP	Matrix metalloproteinase
NF-κB	Nuclear factor-kappa B
pAKT	Phosphorylated Akt
LD	Linear dichroism
PI3K	Phosphatidylinositol 3-Kinase
PI3K/AKT	Phosphatidylinositol 3-Kinase/AKT
UM	Uveal melanoma
