# Supplementary material for: In Vitro Evaluation of Combination Therapy with Doxorubicin and Quercetin for Uveal Melanoma
Source: Curr Issues Mol Biol. 2026 Jun 18;48(6):636. doi: 10.3390/cimb48060636 (PMC13297987; doi:10.3390/cimb48060636)
Supplement: Supplementary file 1 [file cimb-48-00636-s001.zip › Supplementary tables_CIMB.pdf]

Table S1. List of the primer sequences used for qRT-PCR.

Primers	Forward	Reverse
**NF-κB1**	5'-GCAGCACTACTTCTTGACCACC-3'	5'-TCTGCTCCTGAGCATTGACGTC-3'
**p53**	5'-CCTCAGCATCTTATCCGAGTGG-3'	5'-TGGATGGTGGTACAGTCAGAGC-3'
**CYC A**	5'-GGCAAATGCTGGACCCAACACA-3'	5'-TGCTGGTCTTGCCATTCCTGGA-3'

Table S2. List of the antibodies used for Western blots.

Antibody	Origin, catalogue number	Dilution applied
**AKT (pan) (11E7) Rabbit mAb**	Cell Signaling, #4685S	1:1000
**Phospho-AKT (S473) (D9E) Rabbit mAb**	Cell Signaling, #4060S	1:1000
**PI3K p110alpha (C73F8) Rabbit mAb**	Cell Signaling, #4249S	1:1000
**NF-kappaB p65 (D14E12) XP Rabbit mAb**	Cell Signaling, #8242	1:1000
**p53 Rabbit mAb**	Cell Signaling, #9282S	1:1000
**CXCR4 Rabbit polyclonal antibody**	AFFINITY, #AF5279	1:1000
**CXCR6 Rabbit polyclonal antibody**	Abbexa, #abx213070	1:500
**AKT1 antibody [HL1142]**	GeneTex, # GTX636413S	1:1000
**Rabbit polyclonal antibody to MMP9**	Affinity, #AF0220	1:1000
**MMP-2 (D2O4T) Rabbit mAb**	Cell Signaling, #87809	1:1000
**Cyclophillin A Rabbit mAb**	Cell Signaling, #2175S	1:2000
